# Event-related brain response to visual cues in individuals with Internet gaming disorder: relevance to attentional bias and decision-making

**DOI:** 10.1038/s41398-021-01375-x

**Published:** 2021-05-01

**Authors:** Bo-Mi Kim, Jiyoon Lee, A. Ruem Choi, Sun Ju Chung, Minkyung Park, Ja Wook Koo, Ung Gu Kang, Jung-Seok Choi

**Affiliations:** 1 Department of Psychiatry, SMG-SNU Boramae Medical Center, Seoul, 07061 Republic of Korea; 2 Emotion, Cognition and Behavior Research Group, Korea Brain Research Institute, Daegu, 41062 Republic of Korea; 3 Department of Brain and Cognitive Sciences, Daegu Gyeongbuk Institute of Science and Technology, Daegu, 42988 Republic of Korea; 4 Institute of Human Behavioral Medicine, Medical Research Center, Seoul National University, Seoul, 03080 Republic of Korea; 5 Department of Psychiatry and Behavioral Science, Seoul National University College of Medicine, Seoul, 03080 Republic of Korea

**Keywords:** Addiction, Physiology

## Abstract

This study investigated attentional bias toward game-related cues in Internet gaming disorder (IGD) using electrophysiological markers of late positive potential (LPP) and identifying the sources of LPP. In addition, the association between LPP and decision-making ability was investigated. The IGD (*n* = 40) and healthy control (HC; *n* = 39) participants viewed a series of game-related and neutral pictures, while their event-related potentials (ERPs) were recorded. LPPs were calculated as the mean amplitudes between 400 and 700 ms at the centro-parietal (CP3, CP1, Cpz, CP2, and CP4) and parietal (P3, P1, Pz, P2, and P4) electrode sites. The source activations of LPP were estimated using standardized low-resolution brain electromagnetic tomography (sLORETA). In addition, decision-making ability was evaluated by the Cambridge Gambling Task. Higher LPP amplitudes were found for game-related cues in the IGD group than in the HC group. sLORETA showed that the IGD group was more active in the superior and middle temporal gyri, which are involved in social perception, than in the HC group, whereas it was less active in the frontal area. Individuals with IGD have deficits in decision-making ability. In addition, in the HC group, the lower the LPP when looking at the game-related stimuli, the better the quality of decision-making, but not in the IGD group. Enhanced LPP amplitudes are associated with emotional arousal to gaming cues and decision-making deficits in IGD. In addition, source activities suggest that patients with IGD perceive game-related cues as social stimuli. LPP can be used as a neurophysiological marker of IGD.

## Introduction

As the number of Internet gaming users has increased worldwide, new challenges related to Internet gaming are emerging. Excessive use of Internet games leads to psychosocial problems such as decreasing academic and occupational performance^1,2^, interpersonal conflicts, and sleep deprivation^2^. Addressing this social phenomenon, the Diagnostic and Statistical Manual of Mental Disorders fifth edition (DSM-5) recently included the Internet gaming disorder (IGD) in its appendix to encourage further research on the topic^3^. The World Health Organization has also included “Hazardous gaming” and “Gaming disorder” in its 11th revision of the International Classification of Diseases^4^. However, many of the features related to IGD are still inconclusive^5^; therefore, there is a need for further research to explore IGD.

One of the core concepts in recent addiction theories is an incentive motivational process^6–8^. Incentive motivation can be defined as the process of reinforcement through rewarding cognitive and affective states triggered by the perception of conditioned and unconditioned stimuli^9^. Moreover, according to the Incentive-Sensitization Theory^10^, repeated use of drugs or behavior-induced mesolimbic pathway results in incentive salience, which leads to craving for addiction-related cues^11^. Craving is a key phenomenon that causes individuals with addiction to continue and relapse with addictive drugs or addictive behaviors^12^. Craving is the subjective urge to use a drug or to behave in a certain way. In the case of IGD, a craving could be an urge to play a game and approach it^11^. Researchers have suggested that IGD is a behavioral addiction similar to other substance-related addictions^5,13^. Exposure to gaming-related cues can increase their salience and promote craving, thereby facilitating the development of IGD^14,15^.

Attentional bias can be used to measure craving, as increased incentive salience could result in enhanced attention toward addiction-related cues^16–18^. In an electroencephalography (EEG), attentional bias is indicated by an enhancement in the late positive potential (LPP), which is strongly evoked by emotionally salient stimuli^19,20^. The LPP is maximum over centro-parietal regions occurring between 300 and 700 ms following an emotional stimulus^21,22^. Increased LPP amplitude in response to drug-related cues has consistently been observed in various substance-use disorders, such as cocaine-, alcohol-, and nicotine-use disorders^23–25^. Increased LPP amplitudes have also been reported in IGD^26^.

In general, LPP can be triggered by a cue-reactivity task. Cognitive factors such as memory processes and expectations, as well as attentional bias and emotional arousal, have recently been added to the range of cue-reactivity interpretation^27^. The LPP component reportedly reflects cognitive processes such as subjective evaluation^28–30^, activation of motivational systems^30^, and decision-making^31^. Although the source of LPP has been suggested to be in the frontal, occipital, temporal, and parietal area^32,33^, there is still some uncertainty regarding the brain regions associated with LPP and its interpretation. Moreover, literature directly investigating the relationship between LPP amplitude and the source of LPP in IGD is insufficient.

Craving has recently been proposed as an affective state involving behavioral and physiological changes^34^. The neural bases of craving regulation appear to overlap considerably with those underlying the regulation of emotion, including the dorsolateral prefrontal cortex, inferior frontal gyrus, and dorsal anterior cingulate cortex^35,36^. In addition, craving, which is related to the emotional evaluation of addiction-related information, can also influence decision-making processes, favoring choices seeking immediate satisfaction rather than long-term rewards^37–39^.

Decision-making involves many complex cognitive functions, including the process of controlling emotions^40,41^. Hence, there is a possibility that decision-making may be improperly performed if emotional regulation for addiction-related stimulation is impaired. In particular, when an individual with IGD confronts a trigger, it is likely to arouse craving, which in turn leads to inappropriate decision-making (i.e., impulsive behavior).

Another central diagnostic feature of IGD is the compulsive and uncontrolled urge to constantly play games despite negative consequences, such as poor academic performance and impaired social interaction^42^. This impulsive behavior is regarded as a decision-making-related problem and researchers have found that individuals with IGD demonstrate decision-making deficiencies^37,43,44^.

Hence, the aim of this study was to examine deficits in cue-related reactivity using event-related potential (ERP) EEG in individuals with IGD. Furthermore, the source of LPP was investigated to determine their neurobiological traits. In addition, we attempted to examine the relationship between increased LPP and decision-making ability. We hypothesized that individuals with IGD would exhibit increased LPP and show more activation in visual attentional areas in response to game-related stimuli associated with decision-making deficits.

## Materials and methods

### Participants and clinical assessments

The participants in the IGD and healthy control (HC) groups were recruited consecutively from the addiction outpatient clinic at the SMG-SNU Boramae Medical Center, Seoul, Republic of Korea, and via an Internet advertisement from April 2015 to February 2019. An experienced psychiatrist conducted interviews to confirm the diagnosis of IGD using the DSM-5 criteria. A total of 62 patients with IGD were recruited and, after excluding patients with comorbid psychiatric disorders (depressive disorder, *N* = 11; anxiety disorder, *N* = 9; and intellectual disability, *N* = 2), 40 patients with IGD were included in this study. All patients with IGD were medication-naive during assessment and had no comorbid psychiatric disorders. Thirty-nine HC participants were recruited, who played Internet games for <2 h a day and were confirmed to have no past or current psychiatric illness using the Mini-International Neuropsychiatric Interview^45^. For all participants, the Young’s Internet Addiction Test (Y-IAT)^46^ was used to measure the severity of Internet gaming addiction. Beck’s Depression Inventory (BDI-II)^47^ and Beck’s Anxiety Inventory (BAI)^48^ were used to assess depressive and anxiety symptoms, respectively. The participants’ intelligence quotient (IQ) was measured using the abbreviated version of the Korean-Wechsler Adult Intelligence Scale. The exclusion criteria included a lifetime diagnosis of substance abuse or dependence, except for nicotine, neurological disorders, significant head injuries with loss of consciousness, any medical illness with documented cognitive sequelae, sensory impairment, and intellectual disability (IQ < 70). The study protocol was conducted in accordance with the Declaration of Helsinki and was approved by the institutional review board of the SMG-SNU Boramae Medical Center. All participants received detailed information about the study procedure and gave their written informed consent.

### Cue-reactivity task, measure for craving, and EEG recordings

The cue-reactivity task consisted of two types of picture sets as follows: (1) the game-related cues included in-game screen captures of three popular Internet games (i.e., League of Legends, FIFA, and Sudden Attack) and (2) neutral pictures were taken from the neutral category of the International Affective Picture System (IAPS)^49^. Each visual stimulus was matched for size (resolution of 1024 × 768 pixels, 361 × 271 mm, 72 dpi), luminance, brightness, and color. Each category of stimuli (League of Legends, FIFA, Sudden Attack, and Neutral) consisted of seven different pictures and a pseudo-random series of pictures was repeated six times during the task run. The stimulus duration was 3000 ms and the inter-stimulus interval was 2000 ms. The four categories of picture stimuli and sample task sequences are presented in Supplementary Fig. 1.

EEG was performed using a Neuroscan 64-channel Synamps system with a 64-channel Quick-Cap based on the modified 10–20 international system (Compumedics, Charlotte, NC). The electrodes at the mastoid sites served as reference electrodes and the ground electrode was placed between the FPz and Fz electrode sites. The EEG was digitized at a 1000 Hz sampling rate with an online filter of 0.05–100 Hz. Eye-movement artifacts were monitored by recording the vertical and horizontal electro-oculograms using electrodes below and on the outer canthus of the left eye. The resistance at all electrode sites was below 5 kΩ. After completing the EEG recording during the cue-reactivity task, the participants were asked to rate their arousal and valence for all stimuli and their craving in response to the game-related and neutral cues using a visual analog scale ranging from 1 to 10.

### ERP analysis

ERP data were pre-processed using the Curry 7.0 software (Compumedics, Charlotte, NC). The EEG recordings were re-referenced to a common average reference and eye-movement artifacts were reduced using the artifact reduction algorithm in the Curry software^50^. EEG activity was recorded continuously using a 0.1–30 Hz bandpass filter. Events were epoched to 200 ms pre-stimulus and 3000 ms post-stimulus and baseline-corrected using the average pre-stimulus interval voltage. Epochs containing EEG amplitudes in excess of ±100 μV were automatically rejected. Further, the epochs were averaged separately for each stimulus type (game vs. neutral). The LPPs were calculated as the mean values of amplitudes between 400 and 700 ms at the centro-parietal (CP3, CP1, CPz, CP2, and CP4) and parietal (P3, P1, Pz, P2, and P4) electrode sites.

### Source localization of the ERP activity

Standardized low-resolution brain electromagnetic tomography (sLORETA) is widely used for solving the EEG inverse problem to estimate neutral activity in the brain^51,52^. This method assumes that the source activation of one voxel is synchronized with that of the surrounding voxels to calculate a particular solution. In the current study, the source activations of each ERP component were estimated using a realistic head model based on the Montreal Neurological Institute 152 standard template. The source space was restricted to the cortical gray matter, which provided us 6238 voxels with 5 × 5 × 5 mm resolution. The equivalent time window used for ERP peak and mean amplitude analysis was applied to estimations of source activation for each ERP component and condition in both groups using LPP (400–700 ms). All source estimation procedures were performed using the free sLORETA software (http://www.uzh.ch/keyinst/loretaOldy.htm).

### Cambridge gambling task

The Cambridge Gambling Task (CGT)^53^ is part of the Cambridge Neuropsychological Test Automated Battery (Cambridge Cognition Ltd, Cambridge, UK). The CGT measures decision-making and risk-taking outside the learning context. In the CGT, ten red and blue boxes were presented. Participants were required to make a probability judgment on identifying the color box that hid a concealed yellow token and then placing a wager based on their confidence in their decision. Outcome variables used in this task were quality of decision-making (QDM), deliberation time, risk-taking, overall proportion bet, risk adjustment, and delay aversion. In this analysis, we used QDM and risk-taking as variables. The QDM reflects the proportion of trials where the majority color was selected and risk-taking is the mean proportion of the current total points bet when the majority color was selected.

### Statistical analysis

Demographics, clinical characteristics, CGT, and rating scores were compared among the groups using one-way analysis of variance or independent sample *t*-test. The *χ*^2^-test was used for categorical data analysis. Cue effects on the mean LPP amplitudes were analyzed using repeated-measures one-way analysis of covariance (ANCOVA) with the electrode sites (CP3, CP1, CPz, CP2, CP4, P3, P1, Pz, P2, and P4) and the two stimuli (Game and Neutral) as within-subject factors, and the groups (HC and IGD) as between-subjects factors. Group comparisons of the mean LPP amplitudes were performed using repeated-measures ANCOVAs with the ten centro-parietal electrode sites as the within-subject factors and the groups (HC and IGD) as between-subjects factors. The correlations between CGT performance and LPP amplitude were examined using a partial correlation analysis. The covariate was IQ, BDI, and BAI in all ANCOVA and partial correlation analyses. We used the Shapiro–Wilk test to evaluate the normal distribution of data. SPSS version 21(IBM, Inc., Armonk, NY, USA) was used for the statistical analyses. Statistical significance was set at *p* < 0.05.

## Results

### Demographics, clinical characteristics, CGT, and rating for each stimulus

There were no significant differences in terms of age, sex, and smoking (Table 1). However, education, Y-IAT, BDI, BAI, and IQ exhibited significant group differences. The patients with IGD exhibited significantly lower education years [*t*(77) = 2.77, *p* < 0.01] and IQ [*t*(77) = 6.55, *p* < 0.001] compared to the HC group. On the other hand, the patients with IGD scored higher than the HC group in the Y-IAT [*t*(77) = −10.59, *p* < 0.001], BDI [*t*(77) = −8.03, *p* < 0.001], and BAI [*t*(77) = −5.64, *p* < 0.001].

**Table 1:** Demographic and clinical and neurocognitive characteristics of the Internet gaming disorder and healthy control groups.

	Healthy control (*n* = 39)	Internet gaming disorder (*n* = 40)	*X*^*2*^ or *t*	*p*
	Mean (SD)	Mean (SD)		
Demographic characteristics				
Sex (male/female)	29/10	36/4	3.31	0.08
Age (year)	25.13 (3.25)	25.28 (5.56)	−0.14	0.089
Education (year)	14.62 (1.86)	13.43 (1.96)	2.77	0.007^*^
IQ	117.85 (11.14)	101.08 (11.62)	6.55	<0.001^**^
Young’s Internet Addiction Test	30.21 (9.00)	61.03 (15.98)	−10.59	<.001^**^
Time for Internet gaming use at weekday (h/day)	0.29 (0.72)	5.14 (3.75)	−7.92	<.001^**^
Time for Internet gaming use at weekend day (h/day)	0.50 (1.17)	7.96 (10.27)	−4.51	<.001^**^
Smoking (nonsmoker/smoker)	36/3	31/9	3.36	0.63
Number of cigarettes per day for smokers	13.33 (5.77)	18.89 (9.28)	−0.96	0.36
BDI	3.56 (3.83)	17.15 (9.98)	−8.03	<.001^**^
BAI	3.54 (5.07)	15.20 (12.03)	−5.64	<.001^**^
Cambridge Gabling Task				
Quality of decision-making	0.96 (0.05)	0.93 (0.07)	2.24	0.028^***^
Risk-taking	0.49 (0.14)	0.55 (0.14)	−1.83	0.071
Ratings for arousal, valence and craving				
Game stimuli				
Arousal^a^	3.83 (1.68)	4.12 (1.76)	−0.74	0.46
Valence^b^	4.64 (0.78)	5.08 (1.22)	−1.86	0.07
Craving^c^	3.25 (1.78)	4.00 (1.73)	−1.84	0.07
Neutral stimuli				
Arousal^a^	2.58 (1.23)	2.46 (1.30)	0.39	0.70
Valence^b^	5.93 (0.86)	5.72 (0.94)	1.03	0.31
Craving^c^	1.69 (1.17)	1.85 (1.05)	−0.66	0.51

*BAI* Beck’s Anxiety Inventory, *BDI* Beck’s Depression Inventory, *IQ* intelligence quotient.

*P < 0.05, **P < 0.01, ***P < 0.001.

^a^Arousal: extreme calmness (0)–extreme excitement (10).

^b^Valence: extremely negative (0)–extremely positive (10).

^c^Craving: no desire to play game (0)–extreme desire to play game (10).

In terms of CGT, the patients with IGD exhibited significantly lower performance in QDM (*t*(77) = 2.24, *p* < 0.05), indicating deficits in decision-making ability, compared with the HC group. There was no difference between groups in the risk-taking variable. The results are shown in Table 1.

Table 1 also summarizes the results of the ratings for arousal, valence, and craving elicited by each stimulus category. Patients with IGD showed higher levels of valence and craving for the game-related stimulus compared to the HC group, although significances were at a trend level. There was no significant difference in the rating score of arousal for each stimulus between the groups.

### LPP amplitudes

**Fig. 1:**
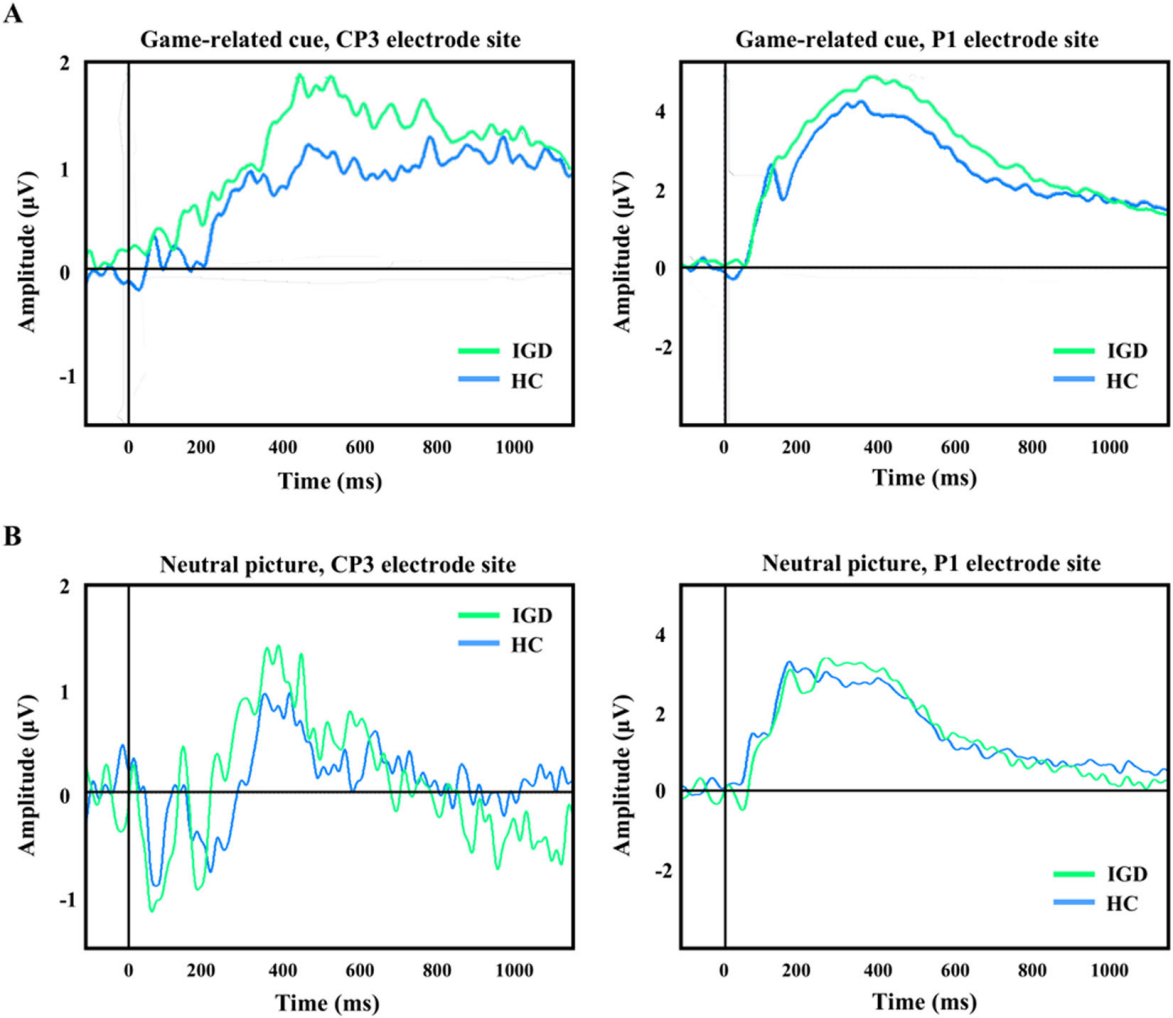
Grand-averaged late positive potential (LPP) waveforms elicited by the game-related and neutral pictures. **A** Grand-averaged LPP waveformselicited by the game-related pictures at the CP3 and P1 electrode sites. **B** Grand-averaged LPP waveforms induced by the neutral pictures at the CP3 and P2 electrode sites.

Figure 1 displays the grand-averaged LPP waveforms at CP3 and P1 elicited by game-related cues. The significant main effect of cues (game and neutral) on mean LPP amplitude [*F*_(1, 77)_ = 4.47, *p* ≤ 0.05] and cues by group interaction [*F*_(1, 77)_ = 5.30, *p* ≤ 0.05] were present. Group effect was not significant for the mean LPP amplitude elicited by neutral IAPS pictures [*F*_(1, 77)_ = 0.68, *p* ≤ 0.41]. Table 2 presents the means (SDs) and the group comparison results for the LPP amplitude at each electrode site. The game-related cues elicited higher LPP amplitudes in the patients with IGD than in the HC participants at the CP3, CP1, and P3 electrode sites. The normal distribution of the variables was confirmed by the Shapiro–Wilk test and the homogeneity of variance between the groups was confirmed.

**Table 2:** Comparison of late positive potentials (LPPs) that averaged between 400 and 700 ms post-stimulus onset across the Internet gaming disorder and healthy control groups.

	Healthy control (*n* = 39)	Internet gaming disorder (*n* = 40)	*F*	*p*
	Mean (SD)	Mean (SD)		
Game stimuli				
CP3 electrode site	0.74 (1.61)	1.42 (1.64)	6.30	0.014^*^
CP1 electrode site	0.70 (1.56)	1.30 (1.86)	5.17	0.026^*^
CPz electrode site	0.60 (1.75)	0.87 (2.06)	0.06	0.43
CP2 electrode site	1.05 (1.78)	0.80 (2.50)	0.04	0.56
CP4 electrode site	1.82 (1.70)	1.52 (2.19)	0.14	0.71
P3 electrode site	3.15 (2.48)	3.96 (3.03)	4.80	0.03^*^
P1 electrode site	3.03 (2.53)	3.50 (3.02)	3.37	0.07
Pz electrode site	2.66 (2.28)	3.02 (2.89)	2.38	0.13
P2 electrode site	2.96 (2.30)	3.31 (3.28)	2.08	0.15
P4 electrode site	3.85 (2.85)	4.12 (3.46)	2.67	0.11
Neutral				
CP3 electrode site	0.48 (2.13)	0.79 (1.96)	0.06	0.81
CP1 electrode site	0.66 (1.98)	0.63 (1.96)	1.00	0.32
CPz electrode site	0.52 (2.22)	0.42 (2.16)	1.33	0.25
CP2 electrode site	0.80 (2.02)	0.67 (2.66)	1.09	0.30
CP4 electrode site	1.24 (2.03)	1.39 (2.53)	0.39	0.53
P3 electrode site	2.59 (2.59)	3.24 (2.54)	0.94	0.34
P1 electrode site	2.72 (2.66)	2.96 (2.60)	0.10	0.75
Pz electrode site	1.93 (2.39)	2.54 (2.72)	0.00	0.98
P2 electrode site	2.23 (2.65)	3.09 (3.13)	0.27	0.61
P4 electrode site	3.06 (2.72)	4.03 (3.34)	0.26	0.61

*P < 0.05.

### sLORETA results

**Fig. 2:**
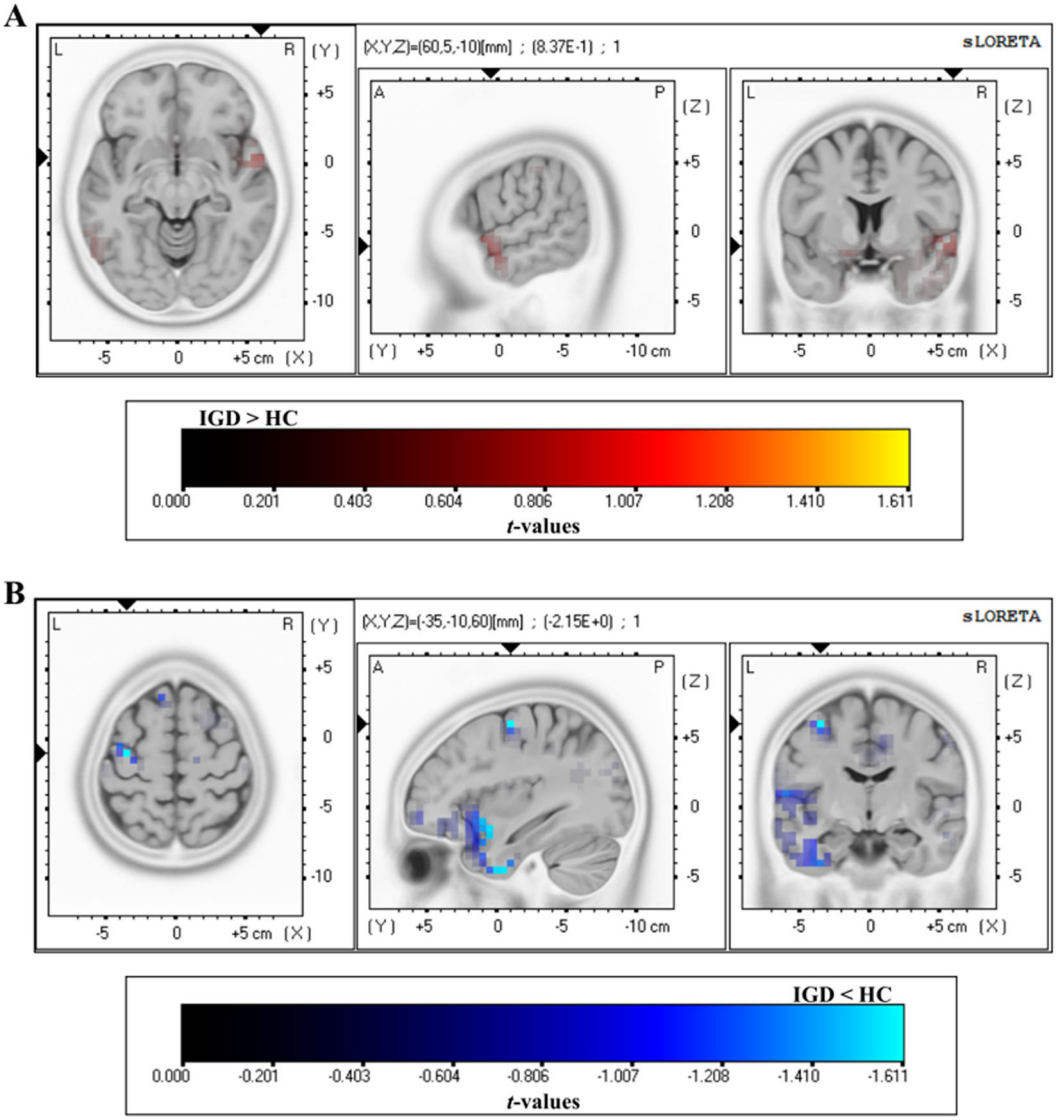
Source localization comparing the current density of the Internet gaming disorder and healthy control groups for the game-related cues in the LPP time range. **A** More activation in the Internet gaming disorder group than the healthy control group and **B** less activation in the Internet gaming disorder group than the healthy control group. The cortical areas show significant differences at the <0.01 level (corrected for multiple comparisons). A, anterior; L, left; P, posterior; R, right. A summary of all significant regions can be found in Supplementary Table S1.

Significant differences were found in the estimated current density between the patients with IGD and the HC group for game-related cues. A significantly higher estimated current density was observed in the patients with IGD compared to the HC group in the superior and middle temporal gyri. A significantly lower estimated current density was observed in patients with IGD compared to the HC group in the frontal, limbic (parahippocampal gyrus, uncus, posterior cingulate, and cingulate gyrus) lobe, cuneus, precuneus, inferior parietal lobule, angular gyrus, and inferior temporal gyrus (see Fig. 2; for a summary of the results, see Supplementary Table S1).

### Relationship between LPP amplitude and decision-making in CGT

**Fig. 3:**
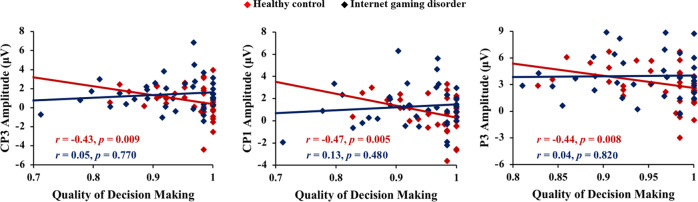
The scatter plot demonstrates the relationship between the quality of decision-making in the Cambridge Gambling Task and LPP elicited by game-related cue in the Internet gaming disorder group and the healthy control group. This correlation was observed significantly only in the healthy control group.

Partial correlation analysis was conducted to investigate the association between LPP amplitude and QDM. Significant differences were found between the IGD and HC groups. In the HC group, there was a negative correlation between the QDM and the LPP amplitude of electrodes CP3 [*r* = −0.43, *p* < 0.01], CP1 [*r* = −0.47, *p* < 0.01], and P3 [*r* = −0.44, *p* < 0.01]. However, there was no significant correlation in the IGD group (see Fig. 3).

## Discussion

In this study, we investigated the characteristics of the neurophysiological markers of craving and attentional bias, and their association with decision-making in individuals with IGD. In particular, we analyzed data only in patients with IGD without comorbid psychiatric disorders to find out core neurophysiological markers of pure IGD. Patients with IGD exhibited increased LPP amplitudes in response to game-related cues, indicating the emotional arousal associated with game-related stimulation. Moreover, the source of brain activity underlying emotional arousal provided that individuals in the IGD group experienced hyperactivation in the superior and middle temporal gyri, which are implicated in social perception, when viewing game-related stimuli. Patients with IGD showed reduced decision-making ability and a loss in the association between LPP amplitudes and QDM, which were conversely observed in the HC group.

Regarding LPP amplitude, the present results are consistent with those of a previous study in which the results indicated an increased LPP amplitude in the IGD group compared to the HC group^26^. Our results further indicated that LPP is a neurophysiological marker for the diagnosis of IGD and attentional bias in individuals with IGD. In a variety of studies, including substance-related studies, increased LPP has been reported to be associated with craving or emotional arousal^21,22,54^. Craving is known to cause addiction-related approach-seeking behavior. If attentional bias occurs after exposure to game-related stimuli and attention cannot be controlled or redirected, the pursuit of addiction behavior occurs. However, our results revealed no significant difference between groups in the emotional assessments scored subjectively by the IGD and HC groups for the presented stimuli. Therefore, considering the characteristics of LPP, we can propose the following: although emotional arousal due to game-related stimuli in individuals with IGD was found to be unrecognized at the conscious level, as they showed blunted or restricted arousal, there were differences between groups with respect to the ERP component. This suggests that the use of objective indicators such as LPP are more important than subjective indicators in the evaluation of craving. In addition, as a patient’s willingness is important for the treatment of addiction^55,56^, awakening self-directed awareness can be the first practical step in clinical treatment for IGD.

Meanwhile, we analyzed the LPP source in the IGD and HC groups when looking at game-related stimuli. LPP sources were predominantly found in the frontal, temporal, and limbic regions. Interesting findings in the present study were that individuals in the IGD group experienced hyperactivation in the superior and middle temporal gyri in response to game-related stimuli. The role of superior and middle temporal regions has been implicated in various aspects of social perception and cognition, including the perception of faces^57^, understanding the behavior of others^58^, and attentional functions such as the control of visual attention^59^. Thus, it can be assumed that game-related stimuli can be interpreted as a social situation for individuals with IGD. The main psychological factor involved in craving is the fear of missing out on certain gaming experiences^60^. The craving for games can be driven by a social desire to maintain competition within a group of peers rather than an intrinsic desire for the game itself^61^. This feature can be more pronounced in online games, in particular, games featuring social cooperative play^62^. Therefore, considering the psychological cause of craving for games and the area activated at the source of LPP, game-related stimuli can be considered a social situation for IGD.

On the other hand, individuals in the IGD group experienced less activation mainly in the prefrontal and limbic regions when viewing game-related stimuli. It is possible that game addiction behavior without experiencing a craving for game-related stimuli at the conscious level is related to the abnormal functioning of the frontal, limbic, and other regions of the brain. The impaired response inhibition and salience attribution (iRISA) model accounts for the contribution of emotional and cognitive processes to addiction^63^. Zilverstand et al.^64^ reviewed previous studies related to this model and classified brain networks related to addiction into 6 types: (a) reward network, (b) habit network, (c) salience network, (d) executive network, (e) self-directed network, and (f) memory network. In terms of the results of this study, we will address three of these six networks^64^. First, the reward network enables subjective evaluation by integrating incentive motivational values and predicting outcomes. Mainly the anterior prefrontal cortex is involved in this process. Second, the salience network is involved in redirecting attentional resources to salient stimuli. In this network, the insular, dorsal anterior cingulate, and inferior parietal lobule are activated. Among them, the insular integrates interoceptive information, and the dorsal anterior cingulate and inferior parietal lobule play a major role in the allocation of attentional control. In substance addiction-related studies, these two network activities were related to self-reported craving and the urge to use^65,66^. Third, self-directed networks, also called default mode networks, include the dorsomedial prefrontal cortex, posterior cingulate cortex, and precuneus. These areas are activated during self-directed, referential cognitive processes and are related to self-focused processing such as self-awareness and self-reflection. In individuals with addiction, the activity of this network was also related to self-reported craving and urge to use. In addition, deficiency in the functioning of this network causes a lack of self-awareness, preventing the network from functioning properly in the self-regulatory and referential process.

Brain areas form networks by interacting with each other and by performing individual roles^67^. These networks can be impaired by local brain regions^68^. Although only the activity of individual brain regions can be confirmed through the source analysis, the fact that the IGD group exhibited activation in different brain regions as compared to the HC group indicates that the brain networks of individuals in the IGD group were also functioning abnormally. Brain regions, including the frontal region, cingulate, and precuneus, which are less active in the IGD group as compared to the HC group, constitute these reward, salience, and self-directed networks. Thus, it is assumed that the reward, salience, and self-directed networks are functioning abnormally. Moreover, one of the most consistent findings in addiction studies is related to abnormalities in the prefrontal cortex. The prefrontal cortex is involved in planning, inhibitory control, and decision-making^69,70^. Individuals with IGD showed lower activation in the frontal region than that in the HC group when looking at game-related stimuli. Thus, its disruption could lead to improper decisions that favor immediate rewards over delayed but more favorable responses, as well as a loss of flexibility in coordinating increased attentional bias to addiction-related stimuli. In particular, the orbitofrontal cortex (OFC) contributes to the representation of reward and punishment, and inhibitory control. Therefore, dysfunction of the OFC is associated with deficiency in the function of response inhibition and impaired decision-making^71–73^.

We observed that individuals with IGD exhibited a greater LPP amplitude than those in the HC group when presented with game-related cues. At the same time, no significant correlation in the IGD group was observed between the increased LPP amplitude and the QDM in CGT. However, it was confirmed that the lower the LPP amplitude in the HC group, the better the QDM in the CGT. Thus, the following can be assumed about the HC group participants: the prefrontal area functions normally, attentional bias is controlled to an appropriate level while viewing game-related cues, and decision-making is appropriate. However, this relationship between the LPP amplitude and decision-making ability was not observed in the IGD group. This suggests that individuals with IGD may not only have control deficits related to playing a game, which is an addictive behavior, but may also face problems in routine decision-making.

This study had several limitations. First, we only investigated the cross-sectional differences between the groups. A longitudinal follow-up of LPP could reveal changes in the cue-related attentional bias on the clinical prognosis. Second, as EEG was performed only during the cue-reactivity task and not during the CGT, direct comparisons of EEG activity in the decision-making process could not be made. Therefore, further research with recorded EEG activity during the decision-making task would allow for a clearer understanding of cognitive processing in individuals with IGD. Finally, other substances, including nicotine, could affect the neurophysiological and cognitive functions in IGD. When we analyzed nonsmoker participants, the current finding remained with no changes. Therefore, we confirmed that nicotine did not affect the current results.

In conclusion, the results of this study suggest that LPP may be cited as a neurophysiological marker of IGD, suggesting the possibility that individuals with IGD might view a game-related stimulus as a social situation. Furthermore, individuals with IGD may struggle with making appropriate decisions in their everyday lives in addition to facing difficulties with managing game-related urges.

## Supplementary information

Supplementary figure legend and table S1

Supplementary figure 1
