# Correction: Event-related brain response to visual cues in individuals with Internet gaming disorder: relevance to attentional bias and decision-making

**DOI:** 10.1038/s41398-021-01424-5

**Published:** 2021-05-26

**Authors:** Bo-Mi Kim, Jiyoon Lee, A. Ruem Choi, Sun Ju Chung, Minkyung Park, Ja Wook Koo, Ung Gu Kang, Jung-Seok Choi

**Affiliations:** 1 Department of Psychiatry, SMG-SNU Boramae Medical Center, Seoul, 07061 Republic of Korea; 2 Emotion, Cognition and Behavior Research Group, Korea Brain Research Institute, Daegu, 41062 Republic of Korea; 3 Department of Brain and Cognitive Sciences, Daegu Gyeongbuk Institute of Science and Technology, Daegu, 42988 Republic of Korea; 4 Institute of Human Behavioral Medicine, Medical Research Center, Seoul National University, Seoul, 03080 Republic of Korea; 5 Department of Psychiatry and Behavioral Science, Seoul National University College of Medicine, Seoul, 03080 Republic of Korea

**Keywords:** Addiction, Physiology

Correction to:

10.1038/s41398-021-01375-x published online 5 May 2021

The original version of this article unfortunately contained a mistake. There was an error in the reference order in the discussion section. The original article has been corrected accordingly.

