# Supplementary material for: Event-related brain response to visual cues in individuals with Internet gaming disorder: relevance to attentional bias and decision-making
Source: Transl Psychiatry. 2021 May 1;11:258. doi: 10.1038/s41398-021-01375-x (PMC8088436; doi:10.1038/s41398-021-01375-x)
Supplement: Supplementary file 1 — Supplementary figure legend and table S1 [file 41398_2021_1375_MOESM1_ESM.docx]

| **Supplementary Table S1.** Significant differences activation of source between the Internet gaming disorder and healthy control groups for the game-related cues. | | | | | |
| --- | --- | --- | --- | --- | --- |
| Brain region | **BA** | **MNI coordinates** | | | ***t*** |
|  |  | **X** | **Y** | **Z** |  |
| Internet gaming disorder > Healthy control | | | | | |
| Middle temporal gyrus | 21 | 60 | 5 | -10 | 0.84 |
| Superior temporal gyrus | 38 | 55 | 5 | -10 | 0.82 |
| Internet gaming disorder < Healthy control | | | | | |
| Precentral gyrus | 6 | -35 | 10 | 60 | -2.15 |
| Parahippocampal gyrus | 34 | -30 | 5 | -20 | -1.84 |
| Postcentral gyrus | 1 | 55 | -20 | 50 | -1.79 |
| Uncus | 28 | -30 | 5 | -25 | -1.70 |
| Angular gyrus | 39 | 50 | -70 | 35 | -1.70 |
| Inferior parietal lobule | 39 | 50 | -65 | 40 | -1.70 |
| Inferior temporal gyrus | 20 | -40 | 0 | -45 | -1.60 |
| Superior temporal gyrus | 38 | -35 | 5 | -15 | -1.55 |
| Middle temporal gyrus | 21 | -40 | 0 | -40 | -1.55 |
| Inferior frontal gyrus | 47 | -30 | 10 | -20 | -1.49 |
| Superior frontal gyrus | 8 | -5 | 25 | 55 | -1.47 |
| Insula | 13 | -30 | 15 | -5 | -1.34 |
| Precuneus | 39 | 45 | -75 | 35 | -1.31 |
| Cuneus | 19 | 20 | -90 | 30 | -1.28 |
| Middle frontal gyrus | 6 | -40 | -5 | 60 | -1.25 |
| Supramarginal gyrus | 40 | -65 | -45 | 30 | -1.23 |
| Medial frontal gyrus | 9 | 5 | 55 | 20 | -1.12 |
| Posterior cingulate | 30 | -5 | -70 | 10 | -1.03 |
| Cingulate gyrus | 32 | -5 | 15 | 45 | -1.03 |
| t-values correspond to p < 0.01  BA: Brodmann Area; MNI = Montreal Neurological Institute | | | | | |

**Supplementary Fig. 1.** A sample of the cue reactivity task sequence.
