# Supplementary figures and images for: Event-related brain response to visual cues in individuals with Internet gaming disorder: relevance to attentional bias and decision-making
Source: Transl Psychiatry. 2021 May 1;11:258. doi: 10.1038/s41398-021-01375-x (PMC8088436; doi:10.1038/s41398-021-01375-x)

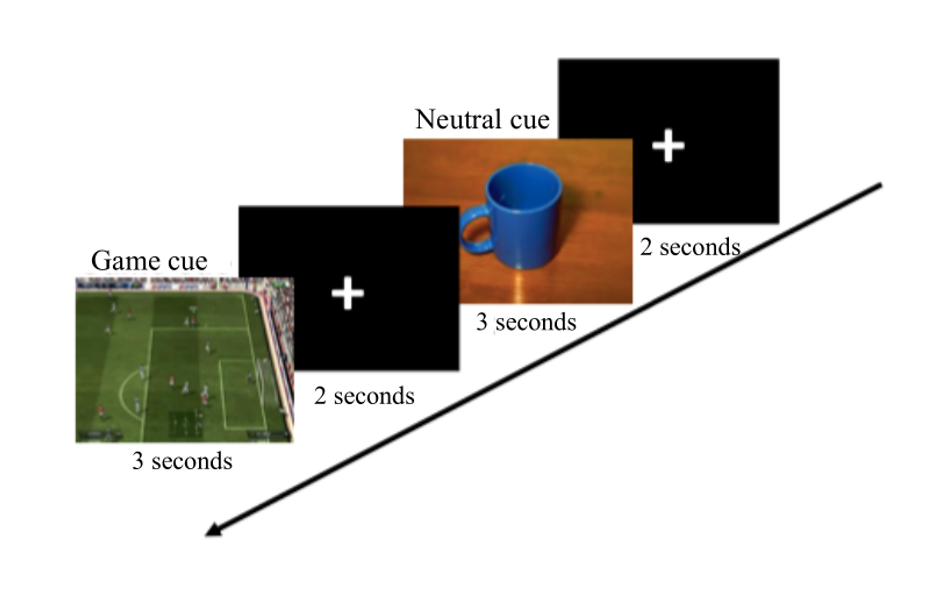

Supplement: Supplementary file 2 — Supplementary figure 1 [file 41398_2021_1375_MOESM2_ESM.tif]
